# Association of cadmium with diabetes in middle-aged residents of abandoned metal mines: the first health effect surveillance for residents in abandoned metal mines

**DOI:** 10.1186/s40557-015-0071-2

**Published:** 2015-08-24

**Authors:** Hee-seung Son, Soo-geun Kim, Byung-seong Suh, Dong-uk Park, Dae-seon Kim, Seung-do Yu, Yeong-seoub Hong, Jung-duck Park, Byung-kook Lee, Jai-dong Moon, Joon Sakong

**Affiliations:** Department of 1Occupational and Environmental Medicine, Kangbuk Samsung Hospital, Medical Center of Sungkyunkwan University School of Medicine, Seoul, Republic of Korea; Department of Environmental Health, Korea National Open University, Seoul, Republic of Korea; Environmental Health Research Department, Environmental Health Research Division, Incheon, Republic of Korea; Department of Preventive Medicine, School of Medicine, Dong-A University, Busan, Republic of Korea; Department of Preventive Medicine, College of Medicine, Chung-Ang University, Seoul, Republic of Korea; Korean Industrial Health Association, Hyesan Bldg., Seoul, Republic of Korea; Department of Preventive Medicine and Public Health, College of Medicine, Chonnam National University Hwasun Hospital, Hwasun, Republic of Korea; Department of Preventive Medicine and Public Health, College of Medicine, Yeungnam University, Daegu, Republic of Korea

**Keywords:** Urine cadmium, Diabetes, Abandoned mine, Glucose, Environmental disorder

## Abstract

**Objective:**

The aim of this study was to determine the association between urinary cadmium (U-cd) concentration and diabetes in middle-aged Korean residents of abandoned mines using the first Health Effect Surveillance for Residents in Abandoned Metal mines (HESRAM).

**Methods:**

This study was cross-sectional study conducted on 719 residents between 40–70 years in 38 abandoned metal mines in Korea. Data was collected by HESRAM from 2008 to 2011. The correlation coefficient of U-cd and fasting blood glucose, odds ratio in urinary cadmium tertiles and diabetes prevalence was analyzed according to the sex category.

**Results:**

The correlation coefficient U-cd concentration and fasting blood glucose was 0.182 in male. Logistic regression analysis in male revealed a third tertile odds ratio of U-cd (2 μg/g creatinine < U-cd) while diabetes prevalence was 1.81 (95 % CI 1.05-3.12) with adjusted age, BMI, smoking and alcohol consumption, region, family income. On the other hand, the odds ratio for third tertile of U-cd (3 μg/g creatinine < U-cd) between diabetes prevalence in female was 1.39 (95 % CI 0.52-3.72) in addition to adjusted menopausal status.

**Conclusions:**

Environmental exposure to cadmium in abandoned mine residents was associated with diabetes in male. Closed monitoring and periodic evaluation of the health effects of chronic environmental exposure on abandoned mines residents will be needed.

## Introduction

Health problems associated with heavy metal exposure have received worldwide concern in the recent past. People living around abandoned metal mines face health risk, as a result of water and soil contamination [1]. Heavy metal pollutants act as endocrine disruptors, when ingested in the body leading to hormonal imbalance [2]. Among heavy metal pollutants, cadmium is the most harmful because it influences several human organs, such as kidney and liver [3, 4].

While diabetic mellitus has become a world-wide health issue [5], its prevalence has been increasing especially in Korea. In 2011, the prevalence of diabetes in the Korean population was 12.4 % among people above 30 years [6, 7].

Pancreatic beta cells dysfunction in releasing insulin is a cause of diabetes [8]. The insufficiency of pancreatic beta cells can be caused by various reasons including genetic vulnerability, ageing, lifestyle, insulin resistance, and environmental factors among others [8]. Some experimental studies on animals showed that cadmium was quickly absorbed bythe pancreatic beta cells in competition with zinc [9, 10].

In this context, it is necessary to consider the association of cadmium with diabetes. A study of hemoglobinA1c (HbA1c) in non-smokers, had positive correlation with cadmium levels in Type II diabetes, impaired fasting glucose (IFG), and impaired glucose tolerance (IGT) patients, however the study had a small sample size [11]. For now, the association of cadmium with diabetes is controversial due to different results [12, 13].

The aim of this study is to determine the association between urinary cadmium (U-cd) concentration and diabetes in middle-aged Korean residents of abandoned mines using the first Health Effect Surveillance for Residents in Abandoned Metal mines (HESRAM). It goes ahead to identify diabetic risk factor linked to cadmium and suggests the basis to control the levels of U-cd.

## Methods

### Study population

HESRAM was performed from 2008 to 2011 in bid to evaluate health effects for residents in 38 abandoned metal mines. The sample size of 2,324 was selected from a population between the ages of 40 and 70 years. Groupsexcluded include: those who did not respond to the questionnaire (*n* = 843), those who did not measure U-cd or blood cadmium (B-cd) concentrations (*n* = 324), and those who did not respond to the period of residence question (*n* = 121).

**Fig. 1:**
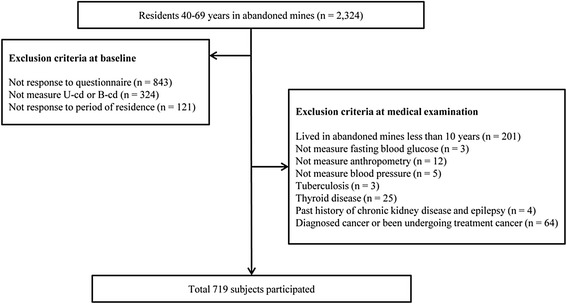
Flow diagram for the selection of study subjects

Out of the remaining 1,036 subjects, exclusion comprised of those who had lived in abandoned mines less than 10 years (*n* = 201), those who did not measure fasting blood glucose (*n* = 3), those who did not measure anthropometry (*n* = 12), those who did not measure blood pressure (*n* = 5), those who had been treated for tuberculosis (*n* = 3), those who had been treated for thyroid disease (*n* = 25), those who had past history of chronic kidney disease and epilepsy (*n* = 4), and those who had been diagnosed with cancer or were undergoing treatment for cancer (*n* = 64). Finally, a total of 719 subjects participated in this study (Fig. 1).

### Sampling and analytical methods

U-cd and B-cd samples were collected in six universities. B-cd was analyzed quantitatively using flameless atomic adsorption spectrometry (AAS). On the other hand, U-cd was analyzed quantitatively using flameless AAS, and graphite furnace attached to atomic absorption spectrophotometer. Both B-cd and U-cd were assayed at Dong-A University. U-cd concentrations were corrected using urinary creatinine concentration. Limit of detection (LOD) of blood cadmium was 0.213 μg/Lin 2010, 0.294 μg/L in2011. For urine cadmium, LOD was 0.0307 μg/L in 2010, 0.0310 μg/L in 2011. Limit of quantification (LOQ) in 2011 was 0.0931 μg/L for blood cadmium, 0.0960 μg/L for urine cadmium.

The criteria for diabetes was defined as the existence of past diabetes history, for cases in which treatment of diabetes resulted in measurement of more than 126 mg/dL in fasting blood glucose according to diabetes diagnostic criteria in American Diabetes Association (ADA) [7].

Blood pressure was measured twice and the average mean was recorded. Fasting blood glucose was measured after fasting at least 8 hours. Height and weight was measured using standardized methods and body mass index (BMI) was calculated using formula. High exposure regions were clustered according to the presence of lead (Pb), zinc (Zn), and copper (Cu) mines and the rest were classified as low exposure regions.

The questionnaire collected information on smoking habits, alcohol consumption, social economic status, and menopausal status. Smoking habits based on the questionnaire was divided into the following categories including: non-smokers, ex-smokers, and current smokers. “Are you smoking a cigarette more than 20 packs so far?“. Alcohol consumption based on the questionnaire was divided into non-drinker, ex-drinker, and current drinker. “ Do you drink? ”Social economic status was categorized into 4 groups according to the family income such as; less than 500,000 won, 500,000-1,000,000 won, 1,000,000-1,500,000 won, above 1,500,000 won. The menopausal status in female was clustered in two groups.

### Data analysis

The U-cd and B-cd concentrations, was used to analyze the correlation coefficient between fasting blood glucose and diabetic risk factor in male and female. Since diabetic drugs lower serum glucose concentration, (avoid using first person tense always use third person) the correlation coefficient analysis was executed in everyone except those eligible for treatment diabetes and serum glucose concentration is less than 126 mg/dL. The chi-square test was performed on the tertile with prevalence diabetes by dividing the U-cd level into third quartile in male and female. Diabetes prevalence odds ratio for the three tertiles of U-cd, was analyzed using multiple logistic regression at 95 % confidence interval. Odds ratio was analyzed in non-adjusted and adjusted models. Statistical analyses were performed using IBM SPSS version 19 (IBM Co., NY, USA).This study was approved by Kangbuk Samsung Hospital Institutional Review Board.

## Result

A total of 719 subjects were used in the study. About 489 (68 %) out of 719 subjects, were male whereas the remaining 230 (32 %) respondents were female with a mean age of 59.1 years for both genders. While, there were no significant age differences between male and female, the BMI was higher for female than male. Blood pressure measurements showed no significant difference between male and female.

B-cd concentration was 1.7 μg/L with no significant differences between male and female, however, the U-cd concentration in female was 2.63 μg/g creatinine, and 2.13 μg/g creatinine in male. It becomes evident that U-cd concentration in female was higher than male. With respect to geometric mean, there was no significant difference in B-cd concentration between male and female.

In the study, the high and low exposure groups were 37.1 % and 62.9 % respectively. Approximately, 34.4 % of the study subjects were non-smokers, 23.6 % ex-smokers, and 42.0 % current smokers. Male were more than female when it comes to current smokers. About 52 % of respondents were current drinkers with male registering a higher percentage than female (Table 1).

**Table 1:** Baseline characteristics of study participants

	Total	Male	Female	*P*-value
	(*n* = 719)	(*n* = 489, 68.0 %)	(*n* = 230, 32.0 %)	
Age, years	59.1 ± 7.5	58.8 ± 7.5	59.8 ± 7.5	0.071
BMI, kg/cm^2^	24.6 ± 3.6	24.2 ± 3.6	25.5 ± 3.5	<0.001
Systolic BP, mmHg	133.5 ± 17.4	133.2 ± 17.4	134.1 ± 17.3	0.514
Diastolic BP, mmHg	79.9 ± 11.0	80.3 ± 11.0	79.1 ± 11.1	0.172
Urine Cadmium, μg/g Cr	2.29 ± 2.20	2.13 ± 2.18	2.63 ± 2.21	0.005
Blood Cadmium, μg/L	1.70 ± 0.97	1.73 ± 0.97	1.64 ± 0.95	0.254
Glucose, mg/dL	110.4 ± 62.0	114.4 ± 69.2	101.9 ± 42.0	0.003
Region†				
High exposure	267 (37.1)	211 (43.1)	56 (24.3)	
Low exposure	452 (62.9)	278 (56.9)	174 (75.7)	<0.001
Smoking status				
Non smoker	247 (34.4)	47 (9.6)	200 (87.0)	
Ex-smoker	170 (23.6)	164 (33.5)	6 (2.6)	
Current smoker	302 (42.0)	278 (56.9)	24 (10.4)	<0.001
Alcohol consumption				
Non-drinker	249 (34.6)	107 (21.9)	142 (61.7)	
Ex-drinker	96 (13.4)	65 (13.3)	31 (13.5)	
Current drinker	374 (52.0)	317 (64.8)	57 (24.8)	<0.001
Family income				
≤500,000 won	342 (47.6)	196 (40.1)	146 (63.5)	
500,000-1,000,000 won	147 (20.4)	105 (21.5)	42 (18.3)	
1,000,000-1,500,000 won	105 (14.6)	85 (17.4)	20 (8.7)	
>1,500,000 won	125 (17.4)	103 (21.1)	22 (9.6)	<0.001
Menopausal status				
Yes	193 (83.9)		193 (83.9)	
No	37 (16.1)		37 (16.1)	

Region was divided by kind of mines; high exposure region was Pb, Zn, Cu mines and low exposure region was otherwise mines

*p*-value was calculated by student t test or chi-square test

BMI, body mass index; BP, blood pressure; Cr, creatinine; AM, arithmetical mean; GM, geometric mean; CI, confidence interval

Table 2 shows the U-cd and B-cd concentrations of study participants, where increase in age, led to the increase in U-cd and B-cd concentrations. Diabetes group had higher U-cd and B-cd concentration than non-diabetic group. B-cd concentration was higher in current smokers and current drinker groups were high.

**Table 2:** Discriptive statistics for urine and blood cadmium levels in study participants

Classification	N(%)	Urine cadmium, μg/g Cr				Blood cadmium, μg/L			
		AM ± SD	*p*	GM(95 % CI)	*p*	AM ± SD	*p*	GM(95 % CI)	*p*
Age group									
40–49	90(12.5)	1.66 ± 1.23		1.55(1.38–1.74)		1.46 ± 0.84		1.35(1.23–1.48)	
50–59	237(33.0)	2.18 ± 1.64		1.91(1.78–2.09)		1.66 ± 0.85		1.51(1.45–1.62)	
60–69	392(54.5)	2.49 ± 2.61	0.004	2.04(1.91–2.14)	0.003	1.79 ± 1.04	0.009	1.66(1.58–1.74)	0.001
Gender									
Male	489(68.0)	2.13 ± 2.18		1.82(1.74–1.91)		1.73 ± 0.97		1.62(1.55–1.70)	
Female	230(32.0)	2.63 ± 2.21	0.005	2.14(2.00–2.34)	<0.001	1.64 ± 0.95	0.254	1.48(1.41–1.58)	0.065
Region									
High exposure	267(37.1)	2.52 ± 2.80		2.04(1.86–2.19)		1.88 ± 0.90		1.74(1.66–1.82)	
Low exposure	452(62.9)	2.15 ± 1.75	0.049	1.86(1.74–1.95)	0.039	1.60 ± 0.99	<0.001	1.48(1.41–1.55)	<0.001
BMI									
<25	412(57.3)	2.37 ± 2.46		2.00(1.86–2.09)		1.79 ± 1.05		1.62(1.55–1.70)	
≥25	307(42.7)	2.17 ± 1.81	0.226	1.86(1.70–2.00)	0.076	1.59 ± 0.83	0.006	1.51(1.41–1.58)	0.026
Diabetes									
Non-diabetes	561(78.0)	2.16 ± 1.70		1.86(1.78–1.95)		1.70 ± 0.95		1.58(1.51–1.62)	
Diabetes	158(22.0)	2.72 ± 3.41	0.049	2.14(1.95–2.40)	0.009	1.70 ± 1.02	0.926	1.58(1.48–1.70)	0.955
Smoking									
Non smoker	247(34.4)	2.51 ± 2.82		2.09(1.91–2.29)		1.52 ± 0.94		1.45(1.35–1.51)	
Ex-smoker	170(27.3)	2.10 ± 1.78		1.78(1.62–1.95)		1.53 ± 0.69		1.45(1.38–1.55)	
Current smoker	302(42.0)	2.21 ± 1.81	0.138	1.91(1.78–2.04)	0.083	1.95 ± 1.06	<0.001	1.77(1.66–1.86)	<0.001
Alcohol consumption									
Non-drinker	248(34.6)	2.57 ± 2.76		2.09(1.91–2.29)		1.69 ± 0.90		1.55(1.45–1.62)	
Ex-drinker	96(13.4)	2.33 ± 1.79		2.04(1.78–2.29)		1.42 ± 0.80		1.38(1.26–1.51)	
Current drinker	374(52.0)	2.09 ± 1.84	0.028	1.78(1.70–1.91)	0.013	1.79 ± 1.03	0.004	1.66(1.55–1.74)	0.009
Family income (won)									
≤500,000	342(47.6)	2.40 ± 2.24		1.95(1.78–2.09)		1.71 ± 1.00		1.58(1.51–1.66)	
500,000–1,000,000	147(20.4)	2.15 ± 1.56		1.95(1.78–2.14)		1.84 ± 0.98		1.70(1.55–1.82)	
1,000,000–1,500,000	105(14.6)	2.12 ± 1.50		1.86(1.66–0.29)		1.60 ± 1.00		1.51(1.38–1.66)	
>1,500,000	125(17.4)	2.29 ± 3.09	0.569	1.91(1.74–2.14)	0.939	1.62 ± 0.80	0.177	1.48(1.38–1.62)	0.164
Menopausal status ( only female)									
Yes	193(83.9)	2.70 ± 1.54		2.19(2.00–2.40)		1.65 ± 0.96		1.51(1.41–1.62)	
No	37(16.1)	2.27 ± 2.32	0.285	2.04(1.66–2.51)	0.534	1.59 ± 0.96	0.736	1.45(1.20–1.70)	0.653

Region was divided by kind of mines; high exposure region was Pb, Zn, Cu mines and low exposure region was otherwise mines

*p*-value was calculated by student t-test or ANOVA

BMI, body mass index; Cr, creatinine; AM, arithmetical mean; SD, standard deviation; GM, geometric mean; CI, confidence interval

Urine cadmium concentration in Diabetes was 2.72 μg/g creatinine and 2.16 μg/g creatinine in non-diabetes. Urine cadmium concentration was higher for diabetic than non-diabetic (*p* = 0.049). There was no significance difference between blood cadmium concentrations in diabetic and non-diabetic (1.70 vs 1.70, *p* = 0.926) (Table 2).

Table 3 shows the correlation between U-cd and fasting blood glucose, and the correlation between U-cd and diabetic risk factors in male and female. The correlation for B-cd was analyzed as well. Out of the 719 subjects, about 684 studied for diabetes treatment had serum glucose level which was less than 126 mg/dL. Nonetheless, 35 subjects were omitted.

**Table 3:** Correlation coefficients between cadmium and clinical variables

	Male(n = 466)		Female(n = 218)	
	Urine cadmium, μg/g creatinine	Blood cadmium, μg/L	Urine cadmium, μg/g creatinine	Blood cadmium, μg/L
Age, year	0.069	0.111*	0.178**	0.110
Systolic BP, mmHg	−0.057	−0.016	−0.094	−0.094
Diastolic BP, mmHg	−0.020	0.040	−0..080	−0.066
BMI, kg/cm^2^	−0.113*	−0.178**	−0.070	−0.074
Glucose, mg/dL	0.182**	0.037	−0.009	−0.067

*p < 0.05

**p < 0.01

While the U-cd concentration was correlated between BMI and fasting blood glucose in male and female, the B-cd concentration in male was correlated between age and BMI. Cadmium concentration was negatively correlated with BMI in male, however, the U-cd concentration in male was positive correlated with fasting blood glucose (Table 3).

According to the mean comparison of age, BMI, and fasting blood glucose in male and female was analyzed not only using ANOVA but also based on the three tertiles of U-cd concentration. The prevalence of diabetes was analyzed with chi-square test (Table 4).

**Table 4:** Comparison of age, glucose, BMI, diabetes prevalence according to the three tertiles of urine cadmium

Male(n = 489)					
	1^st^tertile	2^nd^tertile	3^rd^tertile	*p*- value	Post hoc comparison
	(n = 199)	(n = 151)	(n = 139)		
	(U-Cd ≤1 μg/g Cr)	(1 μg/g Cr < U-Cd ≤2 μg/g Cr)	(2 μg/g Cr < U-Cd)		
Age, year	57.4 ± 7.9	60.1 ± 7.1	59.1 ± 6.8	0.003^a^	I ≠ III^b^
Glucose, mg/dL	105.3 ± 38.9	117.4 ± 66.2	124.3 ± 98.7	0.037^a^	I ≠ III^b^
BMI, kg/cm^2^	24.80 ± 4.00	24.09 ± 3.14	23.36 ± 3.29	<0.001^a^	I ≠ III^b^
Diabetes prevalence	17.1 %	23.8 %	28.8 %	0.010	
Female(n = 176)					
	1^st^tertile	2^nd^tertile	3^rd^tertile	*p* -value	Post hoc comparison
	(*n* = 79)	(*n* = 58)	(*n* = 39)		
	(U-Cd ≤1 μg/g Cr)	(1 μg/g Cr < U-Cd ≤3 μg/g Cr)	(3 μg/g Cr < U-Cd)		
Age, year	58.8 ± 8.4	58.8 ± 7.4	60.7 ± 7.0	0.408^a^	
Glucose, mg/dL	102.7 ± 49.1	102.8 ± 41.8	106.0 ± 39.5	0.923^a^	
BMI, kg/cm^2^	25.86 ± 3.26	25.83 ± 3.38	24.48 ± 3.15	0.074^a^	
Diabetes prevalence	20.3 %	17.2 %	30.8 %	0.282	

^a^Statistical significances were tested by one way analysis of variances among groups

^b^The same letters indicate non-significant difference between groups based on Tukey’s multiple comparison test

U-cd concentration in the study was not correctly divided into three tertile because many cases represented the same U-cd concentration. In male, the first tertile was ≤1 μg/g creatinine in U-cd, while the second tertile was 1 μg/g creatinine < U-cd ≤ 2 μg/g creatinine. The third tertile was >2 μg/g creatinine in U-cd. In female, the first tertile was ≤ 1 μg/g creatinine in U-cd, while the second tertile was 1 μg/g creatinine < U-cd ≤ 3 μg/g creatinine, and the third tertile was > 3 μg/g creatinine in U-cd.

Age, glucose, BMI showed differences in each tertile in male with no difference in female. According to the increase in tertile, the prevalence of diabetes increased significantly in male but there was no statistically significant increase in female.

Based on the first tertile, Table 5 showed odds ratio and 95 % confidence interval for the prevalence of diabetes to the second and third tertile of U-cd concentration in male and female. Three models used for analysis include, Model 1 was not adjusted for confounding factor, Model 2 was adjusted for age And Model 3 was adjusted for age, sex, BMI, smoking habits, alcohol consumption, region, family income in male. Model 3was additionally adjusted for menopausal status in female.

**Table 5:** Non–adjusted and adjusted odds ratio and 95 % CI of prevalent diabetes according to the three tertiles of urine cadmium

Male (*n* = 489)			
	1^st^tertile	2^nd^tertile	3^rd^tertile
	(*n* = 199)	(*n* = 151)	(*n* = 139)
	(U-Cd ≤1 μg/g Cr)	(1 μg/g Cr < U-Cd ≤2 μg/g Cr)	(2 μg/g Cr < U-Cd)
Model 1	Reference	1.52 (0.90–2.57)	1.96 (1.17–3.30)
Model 2	Reference	1.42 (0.83–2.42)	1.89 (1.12–3.19)
Model 3	Reference	1.42 (0.83–2.45)	1.81(1.05–3.12)
Female (*n* = 176)			
	1^st^tertile	2^nd^tertile	3^rd^tertile
	(*n* = 79)	(*n* = 58)	(*n* = 39)
	(U–Cd ≤1 μg/g Cr)	(1 μg/g Cr < U–Cd ≤3 μg/g Cr)	(3 μg/g Cr < U–Cd)
Model 1	Reference	0.82 (0.34–1.97)	1.75 (0.73–4.19)
Model 2	Reference	0.82 (0.34–1.97)	1.72 (0.72–4.15)
Model 3*	Reference	0.66 (0.25–1.73)	1.39 (0.52–3.72)

Model 1: non–adjusted

Model 2: adjusted for age

Model 3: adjusted for age, BMI, smoking, alcohol consumption, region, family income

Model 3*: adjusted for age, BMI, smoking, alcohol consumption, region, family income, menopausal status

The odds ratio in male was 1.52 in the second tertile, however, it was not statistically significant at model 1. The odds ratio was 1.96 in third tertile and statistically significant. Similar to model 1, model 2, like was not statistically significant in the second tertile. However, the odds ratio in the third tertile was 1.89 and statistically significant. Similarly model 3 showed no statistical significance in second tertile. Nonetheless, the odds ratio in the third tertilewas 1.81 and statistically significant. In females, there was no statistically significant in all models.

## Discussion

Chronic exposure to cadmium resulted from smoking and environmental exposure [14, 15]. Cadmium does not exist solely by mining activities. It is produced as a by-product in the process of mining and melting zinc, copper, and lead [16].

B-cd concentration was 1.70 μg/L in this study, nevertheless if compared to the study based on the Korea National Health and Nutrition Examination Survey (KNHANES), the Korea population’s blood cadmium concentration was 1.16 μg/L in diabetes and 1.10 μg/L in non-diabetes [13]. The B-cd level of residents in abandoned mines was higher than Korean population. U-cd concentration was 2.29 μg/g creatinine in the study, this was less than biologic exposure indices (BEI), 5 μg/g creatinine. Moreover, in the Korean lead worker study, urine cadmium concentration was 0.98 μg/g creatinine [17], while 1.93 μg/g creatinine was evident in a Japenese population study [18]. We found that B-cd level and U-cd level of residents in abandoned mines was relatively higher than other population.

B-cd level reflects recent exposure, U-cd level reflects a relatively past exposure. If low exposure of cadmium continues, U-cd reflects the accumulation in body amounts [19, 20]. For this reason, in order to determine the effect on chronic cadmium exposure, U-cd level was used in the study.

Diabetes prevalence in residents of abandoned mines was 22 %, this was higher than the prevalence of diabetes for Koreans over 30 years, 12.4 % [7]. Prevalence of diabetes in the Urban area was at 14.5 % [21]. The study population was more than 40 years because diabetes is prevalent in this demographics. Moreover, the prevalence of diabetes in urban areas was higher than in rural areas in developing countries with urbanization trends, however, rural areas showed higher prevalence of diabetes than urban areas in developed countries [22, 23]. Finally, the study was limited to residents in abandoned mines, and its results are difficult to generalize the entire Korean population.

Previous studies that related cadmium with diabetes showed no cadmium concentration [13]. Nevertheless previous studies had disadvantage in using B-cd level. In a foreign study, U-cd level in diabetic group was higher than non-diabetic group [24]. In other studies, increased U-cd level, corresponded with increased fasting blood glucose and prevalence of type II diabetes [12]. In this study, as chronic exposure marker, U-cd concentration was increased; the prevalence of diabetes was higher.

There was no statistically significant relationship between cadmium and diabetes in female. In a Swedenish female prospective study, there were no associations between B-cd or U-cd concentrations and the prevalence or incidence of type II diabetes or impaired glucose tolerance [25]. A study conducted on the northwestern Thailand population revealed no significant association between U-cd and diabetes [26]. These inconsistent findings might be due to different study subjects and methodological limitations such as small study group size, suboptimal subject selection, and lack of adjustment for other potential risk factors. Further study may be useful to infer association cadmium exposure and diabetes in female.

Effects of cadmium are as follows in the development of diabetes. Cadmium is absorbed directly to pancreatic beta cells involved in insulin secretion. In animal experimental study, cadmium was rapidly accumulated in the pancreatic beta cell in mice with hyperglycemia and obese [27]. Cadmium accumulation induced degeneration, necrosis, and weak degranulation in the pancreatic β-cell [28, 29]. Cadmium activates gluconeogenesis on the liver cell and impaires insulin receptor [30]. Furthermore, cadmium Adipocyte in past exposure to cadmium decreased glucose transporter expression and glucose transport activity in cell study, induce a dose-dependent reduction in GLUT4 protein and mRNA expressions [31].

The present study revealed an association of Cadmium with Diabetes in the middle aged residents of Abandoned Metal Mines in male. Prevalence of diabetes was increased in >2 μg/g creatinine in U-cd concentration in male. Moreover, there was a significant association between environmental exposure and disease in residents of abandoned mines. Other studies had associated low concentration cadmium with health effect [12, 32]. Thus, even if there is low-concentration exposure under biological exposure standards, chronic exposure is significant in that it may affect the development of the disease.

While the study had limitations, it utilized type II diabetes to identify association of cadmium with diabetes. On the other hand, HESRAM did not distinguish between type I and type II diabetes in the questionnaire. Thus, the prevalence of diabetes was higher, which may have affected the association of cadmium with diabetes. The study was an attempt to mitigate the impact of the prevalence of typeIdiabetes using the age of 40 years or older. Information on the effect of exposure of other heavy metals is insufficient. In future studies, it will be necessary to study other heavy metals that can affect diabetes together.

Hip/waist ratio has been used to reflect obesity rather than BMI [33]. Because the present study had no data to hip and waist circumference, hip/waist ratio was replaced with BMI. Thus, future studies, should tackle the BMI issue. One of the problems with the questionnaire was the failure to quantify about smoking and alcohol consumption. There was no association between U-cd and diabetes in female. The reason for inconsistence with other studies may be due to a small sample size, selection bias and inadequate information. It will be necessary for further studies to modify the methodology. In male, the association between U-cd and diabetes was observed.

Finally, as a cross-sectional study, it had limits. Additional study design on the topic of whether exposure to chronic cadmium in the development of diabetes is influential would be necessary. However, in spite of these limitations, the biological relation of cadmium and diabetes was confirmed via conventional animal experiments and epidemiological studies. Conclusively there is association U-cd concentration and diabetes.

## Conclusion

Even though heavy metal mines have been closed, residents in abandoned mines run the risk of chronic exposure particulalry when exposed to chronic cadmium, not exceeding biological exposure limits. Results of the present study confirm that low concentration of U-cd is associated with diabetes, and may be act as a risk factor for development of diabetes in male. Therefore, it is necessary to observe regular health impact assessment of chronic environmental exposure of residents in abandoned mines. Further studies would be necessary to female.
